# Supplementary material for: Heat adaptation of phage T7 under an extended genetic code
Source: Virus Evol. 2021 Dec 1;7(2):veab100. doi: 10.1093/ve/veab100 (PMC8923235; doi:10.1093/ve/veab100)
Supplement: veab100_Supp [file veab100_supp.zip › Sequencing_Analysis.zip!/Sequencing_Analysis/S1/evidence/RA_29.html]

BRESEQ :: Evidence


| Read alignment evidence... | | | | | | | | | | | |
| --- | --- | --- | --- | --- | --- | --- | --- | --- | --- | --- | --- |
|  | seq id | position | | ref | new | freq | score (cons/poly) | reads | annotation | genes | product |
| \* | NC\_001604.1 | 29,697 | 0 | C | T | 72.4% | 4495.6 / inf | 3542 | intergenic (–/–) | *–/–* | –/– |
| Reads supporting (aligned to +/- strand):  **ref** base C (480/484);  **new** base T (1298/1231);  **total** (1804/1738) | | | | | | | | | | | |
| Fisher's exact test for biased strand distribution *p*-value = 4.27e-01 | | | | | | | | | | | |
| Kolmogorov-Smirnov test that lower quality scores support variant *p*-value = 1.99e-01 | | | | | | | | | | | |

Only 100 of 3545 total aligned reads displayed.  

`AAGTTCAGAATCAGATGAACGCATGGACGAAAGCTCAGGCCAAGGCTCTGGACGATTCCATGAAGTCAATGAACAAACTTGACGTAATCGACAAGCAATTCCAGAAGCGAATCAACGGTGAGTGGGTCTCAACGGATTTTAAGGATATGCCAGTCAACGAGAACACTGGTGAGTTCAAGCATAGCGATATGGTTAACTACGCCAATAAGAAGCTCGCTGAGATTGACAGTATGGACATTCCAGACGGTGCCAAGGATGCTATGAAGTTGAAGTACCTTCAAGCGGACTCTAAGGACGGAGCATTCCGTACAGCCATCGGAACCATGGTCACTGACGCTGGTCAAGAGTGGTCTGCCGCTGTGATTAACGGTAAGTTACCAGAACGAACCCCAGCTATGGATGCTCTGCGCAGAATCCGCAATGCTGACCCTCAGTTGATTGCTGCGCTATACCCAGACCAAGCTGAGCTATTCCTGACGATGGACATGATGGACAAGCAGGGTATTGACCCTCAGGTTATTCTTGATGCCGACCGACTGACTGTTAAGCGGTCCAAAGAGCAACGCTTTGAGGATGATAAAGCATTCGAGTCTGCACTGA  >  NC_001604.1/29397‑29996`  

                                                                                                                                                                                                                                                                                                            `|`                                                                                                                                                                                                                                                                                                             
      `aGAATCAGATGAACGCATGGACGAAAGCTCAGGCCAAGGCTCTGGACGATTCCATGAAGTCAATGAACAAACTTGACGTAATCGACAAGCAATTCCAGAAGCGAATCAACGGTGAGTGGGTCTCAACGGATTTTAAGGATATGCCAGTCAACGAGAACACTGGTGAGTTCAAGCATAGCGATATGGTTAACTACGCCAATAAGAAGCTCGCTGAGATTGACAGTATGGACATTCCAGACGGTGCCAAGGATGCTATGAAGTTGAAGTACCTTCAAGCGGACTCTAAGGGCGGAGCATTCCg                                                                                                                                                                                                                                                                                                       <  1:347294/301‑1 (MQ=255)`  
       `gAATCAGATGAACGCATGGACGAAAGCTCATGCCAAGGATCTGGACGATTCCATGAAGTCAATGAACAAACTTGACGTAATCGACAAGCAATTCCAGAAGCGAATCAACGGTGAGTGGGTCTCAACGGATTTTAAGGATATGCCAGTCAACGAGAACACTGGTGAGTTCAAGCATAGCGATATGGTTAACTACGCCAATAAGAAGCTCGCTGAGATTGACAGTATGGACATTCCAGACGGTGCCAAGGATGCTATGAAGTTGAAGTACCTTCAAGCGGACTCTAAGGACGGAGTATTCCGt                                                                                                                                                                                                                                                                                                      <  1:305334/301‑1 (MQ=255)`  
       `gAATCAGATGAACGCATGGACGAAAGCTCAGGCCAAGGCTCTGGACGATTCCATGAAGTCAATGAACAAACTTGACGTAATCGACAAGCAATTCCAGAAGCGAATCAACGGTGAGTGGGTCTCAACGGATTTTAAGGATATGCCAGTCAACGAGAACACTGGTGAGTTCAAGTATAGCGATATGGTTAACTACGCCAATAAGAAGCTCGCTGAGATTGACATTATGGACATTCCAGACGGTGTCAAGGATGCTATGAAGTTGAAGTACCTTCAAGCGGACTCTACGGACGGAGCATTCCGt                                                                                                                                                                                                                                                                                                      >  1:105477/1‑301 (MQ=255)`  
       `acagcAGATGAACGCATGGACGAAAGCTCAGGCCAAGGCTCTGGACGATTCCATGAAGTCAATGAACAAACTTGACGTAATCGACAAGCAATTCCAGAAGCGAATCAACGGTGAGTGGGTCTCAACGGATTTTAAGGATATGCCAGTCAACGAGAACACTGGTGAGTTCAAGCATAGCGATATGGTTAACTACGCCAATAAGAAGCTCGCTGAGATTGACAGTATGGACATTCCAGACGGTGCCAAGGATGCTATGAAGTTGAAGTACCTTCAAGCGGACTCTAAGGACGGAGTATTCCGt                                                                                                                                                                                                                                                                                                      <  1:183655/297‑1 (MQ=255)`  
       `aaaTCAGATGAACGCATGGACGAAAGCTCAGGCCAAGGCTCTGGACGATTCCACGAAGTCAATGAACAAACTTGACGTAATCGACAAGCAATTCCAGAAGCGAATCAACGGTGAGTGGGTCTCAACGGATTTTAAGGATATGCCAGTCAACGAGAACACTGGTGAGTTCAAGCATAGCGATATGGTTAACTACGCCAATAAGAAGCTCGCTGAGATTGACAGTATGGACATTCCAGACGGTGCCAAGGATGCTATGAAGTTGAAGTACCTTCAAGCGGACTCTAAGGACGGAGTATTCCGt                                                                                                                                                                                                                                                                                                      <  1:118564/300‑1 (MQ=255)`  
       `aaaTCAGATGAACGCATGGACGAAAGCTCAGGCAAAGGCTCTGGACGATTCCATGAAGTCAATGAACAAACTTGACGTAATCGACAAGCAATTCCAGAAGCGAATCAACGGTGAGTGGGTCTCAACGGATTTTAAGGATATGCCAGTCAACGAGAACACTGGTGAGTTCAAGCATAGCGATATGGTTAACTACGCCAATAAGAAGCTCGCTGAGATTGACAGTATGGACATTCCAGACGGTGCCAAGGATGCTATGAAGTTGAAGTACCTTCAAGCGGACTCTAAGGACGGAGTATTCCGt                                                                                                                                                                                                                                                                                                      <  1:423720/300‑1 (MQ=255)`  
              `aggAACGCATGGACGAAAGCTCAGGCCAAGTCTCTGGACGATTCCATGAAGTCAATGAACAAACTTGACGTAATCGACAAGCAAGTCCAGAAGCGAATCAACGGTGAGTGGGTCTCAACGGATTTTAAGGATATGCCAGTCAACGAGAACACTGGTGAGTTCAAGTATAGCGATATGGTTAACTACGCCAATAAGAAGCTCGCTGAGATTGACAGTATGGACATTCCAGACGGTGCCAAGGATGCTATGAAGTTGAAGTACCTTCAAGCGGACTCTAAGGACGGAGCATTCCGTACAGCCa                                                                                                                                                                                                                                                                                               <  1:597543/299‑1 (MQ=255)`  
                        `ggACGAAAGCTCAGGCCAAGGCTCTGGACGATTCCATGAAGTCAATGAACAAACTTGACGTAATCGACAAGCAATTCCAGAAGCGAATCAACGGTGAGTGGGTCTCAACGGATTTTAAGGATATGCCAGTCAACGAGAACACTGGTGAGTTCAAGCATAGCGATATGGTTAACTACGCCAATAAGAAGCTCGCTGAGATTGACATTATGGACATTCCAGACGGTGCCAAGGATGCTATGAAGTTGAAGTACCTTCAAGCGGACTCTAAGGACGGAGTATTCCGTACAGCCATCGGAACCAt                                                                                                                                                                                                                                                                                     >  1:549091/1‑301 (MQ=255)`  
                         `gACGAAAGCTCAGGCCAAGGCTCTGGACGATTCCATGAAGTCAATGAACAAACTTGATGTAATCGACAAGCAATTCCAGAAGCGAATCAACGGTGAGTGGGTCTCAACGGATTTTAAGGATATGCCAGTCAACGAGAACACTGGTGAGTTCAAGCATAGCGATATGGTTAACTACGCCAATAAGAAGCTCGCTGAGATTGACAGTATGGACATTCCAGATGGTGCCAAGGATGCTATGAAGTTGAAGTACCTTCAAGCGGACTCTAAGGGCGGAGCATTCCGTACAGCCATCGGAACCATg                                                                                                                                                                                                                                                                                    >  1:591586/1‑301 (MQ=255)`  
                         `gACGAAAGCTCAGGCCAAGGCTCTGGACGATTCCATGAAGTCAATGAACAAACTTGACGTAATCGACAAGCAATTCCAGAAGCGAATCAACGGTGAGTGGGTCTCAACGGATTTTAAGGATATGCCAGTCAACGAGAACACTGGTGAGTTCAAGCATAGCGATATGGTTAACTACGCCAATAAGAAGCTCGCTGAGATTGACAGTATGGACATTCCAGACGGTGCCAAGGATGCTATGAAGTTGAAGTACCTTCAAGTGGACTCTAAGGACGGAGTATTCCGTACAGCCATCGGAACCATg                                                                                                                                                                                                                                                                                    >  1:198830/1‑301 (MQ=255)`  
                                    `aagCCAAAGCTCTGGAAGAATCAAATAAGTCAATTAACAATCTTGACGTAATCGACAAGCAATTCCAGAAGCGAATAAACGGTGAGTGGGTCTCAACGGATTTTAAGGATAAGCCATTCAACGAGAACACTGGTGAGTTCAAGCATAGCGATATGGTGAACGACGCCAATAAGAAGCTCGCTGAGATTGACAGTATGGACATTCCAGACGGTGCCAAGGATGCTATGAAGTTGAAGTACCTTCAAGCGGACTCTAAGGACGGAGTATTCCGTACAGCCATCGGAACCATGGTCACTGACGc                                                                                                                                                                                                                                                                         <  1:70511/299‑1 (MQ=255)`  
                                    `aGGCGAAGGCTCTGGACGATTCCAGGAAGTCAATGAACAAACTTGACGTAATCGACAAGCAATTCCAGAAGCGAATCAACGGTGAGTGGGTCTCAACGGATTTTAAGGATATGCCAGTCAACGAGAACACTGGTGAGTTCAAGTATAGCGATATGGTTAACTACGCCAATAAGAAGCTCGCTGAGATTGACAGTATGGACATTCCAGACGGTGCCAAGGATGCTATGAAGTTGAAGTACCTTCAAGCGGACTCTAAGGACGGAGCATTCCGTACAGCCATCGGAACCATGGTCACTGACGc                                                                                                                                                                                                                                                                         <  1:75929/301‑1 (MQ=255)`  
                                    `aGGCCAAGGCTCTGGACGATTCCATGAAGTCAATGAACAAACTTGACGTAATCGACAAGCAATTCCAGAAGCGAATCAACGGTGAGTGGGTCTCAACGGATTTTAAGGATATTCCAGTCAACGAGAACACTGGTGAGTTCAAGCATAGCGATATGGTTAACTACGCCAATAAGAAGCTCGCTGAGATTGACAGTATGGACATTCCAGACGGTGCCAAGGATGCTATGAAGTTGAAGTACCTTCAAGCGGACTCTAAGGACGGAGTATTCCGTACAGCCATCGGAACCATGGTCACTGACGc                                                                                                                                                                                                                                                                         <  1:497864/301‑1 (MQ=255)`  
                                                 `ggACGAGTCAAGGAATACAATTAACAAACTTGACGTAATCGACAATCAAGTCCAGAAGAGAATCAACGGTGAGTGGGTCTCAACGGATTTTAAGGATATGCCAGTCAACGAGAACACTGGTGAGTTCAAGCATAGCGATATGGTTAACTACGCCAATAAGAAGCTCGCTGAGATTGACAGTATGGACATTCCAGACGGTGCCAAGGATGCTATGAAGTTGAAGTACCTTCAAGCGGACTCTAAGGACGGAGTATTCCGTACAGCCATCGGAACCATGGTCACTGACGCTGGTCAAGAGTgg                                                                                                                                                                                                                                                            <  1:392838/301‑1 (MQ=255)`  
                                                      `aTTCCATGAAGTCAATGAACAAACTTGACGTAATCGACAAGCAATTCCAGAAGCGAATCAACGGTGAGTGGGTCTCAACGGATTTTAAGGATATGCCAGTCAACGAGAACACTGGTGAGTTCAAGCATAGCGATATGGTTAACTACGCCAATAAGAAGCTCGCTGAGATTGACAGTATGGACATTCCAGACGGTGCCAAGGATGCTATGAAGTTGAAGTACCTTCAAGCGGACTCTAAGGACGGAGTATTCCGTACAGCCATCGGAACCATGGTCACTTACGCTGGTCAAGAGTGGTCTGc                                                                                                                                                                                                                                                       >  1:204414/1‑301 (MQ=255)`  
                                                        `tCCATGAAGTCAATGAACAAACTTGACGTAATCGACAAGCAATTCCAGAAGCGAATCAACGGTGAGTGGGTCTCAACGGATTTTAAGGATATTCCAGTCAACGAGAACACTGGTGAGTTCAAGCATAGCGATATGGTTAACTACGCCAATAAGAAGCTCGCTGAGATTGACAGTATGGACATTCCAGACGGTGCCAAGGATGCTATGAAGTTGAAGTACCTTCAAGCGGACTCTAAGGACGGAGTATTCCGTACAGCCATCGGAACCATGGTCACTGACGCTGGTCAAGAGtctgtctctt                                                                                                                                                                                                                                                     >  1:88214/1‑292 (MQ=255)`  
                                                        `tCCATGAAGTCAATGAACAAACTTGACGTAATCGACAAGCAATTCCAGAAGCGAATCAACGGTGAGTGGGTCTCAACGGATTTTAAGGATATGCCAGTCAACGAGAACACTGGTGAGTTCAAGCATAGCGATATGGTTAACTACGCCAATAAGAAGCTCGCTGAGATTGACAGTATGGACATTCCAGACGGTGCCAGGGATGCTATGAAGTTGAAGTACCTTCAAGCGGACTCTAAGGCCGGAGTATTCAGTACAGCCATCGGAACCATGGTCACTGACGAGGGTCAAGAGTGGTCTGccc                                                                                                                                                                                                                                                     >  1:653923/1‑300 (MQ=255)`  
                                                              `aaGTCAATGAACAAACTTGACGTAATCGACAAGCAATTCCAGAAGCGAATCAACGGTGAGTGGGTCTCAACGGATTTTAAGGATATGCCAGTCAACGAGAACACTGGTGAGTTCAAGTATAGCGATATGGTTAACTACGCCAATAAGAAGCTCGCTGAGATTGACAGTATGGACATTCCAGACGGTGCCAAGGATGCTATGAAGTTGAAGTACCTTCAAGCGGACTCTAAGGACGGAGCATTACGTACAGCCATCGGAACCATGGTCACTGACGCTGGTCAAGAGCTGTCtcttatacaca                                                                                                                                                                                                                                               >  1:567153/1‑291 (MQ=255)`  
                                                              `aaGTCAATGAACAAACTTGACGTAATCGACAAGCAATTCCAGAAGCGAATCAACGGTGAGTGGGTCTCAACGGATTTTAAGGATATGCCAGTCAACGAGAACACTGGTGAGTTCAAGCATAGCGATATGGTTAACTACGCCAATAAGAAGCTCGCTGAGATTGACAGTATGGACATTCCAGACGGTGCCAAGGATGCTATGAAGTTGAAGTACCTTCAAGCGGACTCTAAGGACGGAGTATTCCGTACAGCCATCGGAACCATGGTCACTGACGCTGGTCAAGAGTGGTCTGccctgtctc                                                                                                                                                                                                                                               >  1:417847/1‑294 (MQ=255)`  
                                                                `gTCAATGAACAAACTTGACGTAATCGACAAGCAATTCCAGAAGCGAATCAACGGTGAGTGGGTCTCAACGGATTTTAAGGATATGCCAGTCAACGAGAACACTGGTGAGTTCAAGCATAGCGATATGGTTAACTACGCCAATAAGAAGCTCGCTGAGATTGACAGTATGGACATTCCAGACGGTGCCAAGGATGCTATGAAGTTGAAGTACCTTCAAGCGGACTCTAAGGACGGAGTATTCCGTACAGCCATCGGAACCATGGTCACTGACGCTGGTCAAGAGTGGTCTGccctgtctctt                                                                                                                                                                                                                                             >  1:248466/1‑292 (MQ=255)`  
                                                                   `aaTGAACAAACTTGACGTAATCGACAAGCAATTCCAGAAGCGAATCAACGGTGAGTGGGTCTCAACGGATTTTAAGGATATGCCAGTCAACGAGAACACTGGTGAGTTCAAGCATAGCGATATGGTTAACTACGCCAATAAGAAGCTCGCTGAGATTGACAGTATGGACATTCCAGACGGTGCCAAGGATGCTATGAAGTTGAAGTACCTTCAAGCGGACTCTAAGGACGGAGTATTCCGTACAGCCATCGGAACCATGGTCACTGACGCTGGTCAAGAGTGGTCTGCCGCTGTGATTAAc                                                                                                                                                                                                                                          <  1:194510/301‑1 (MQ=255)`  
                                                                           `aatgtgtataagagacagaGCAATTCCAGAAGCGAATCAACGGTGAGTGGGTCTCAACGGATTTTAAGGATATGCCAGTCAACGAGAACACTGGTGAGTTCAAGCATAGCGATATGGTTAACTACGCCAATAAGAAGCTCGCTGAGATTGACAGTATGGACATTCCAGACGGTGCCAAGGATGCTATGAAGTTGAAGTACCTTCAAGCGGACTCTAAGGACGGAGTATTCCGTACAGCCATCGGAACCATGGTCACTGACGCTGGTCAAGAGTGGTCTGCCGCTGTGATTAACGGTAAGtt                                                                                                                                                                                                                                  <  1:492505/283‑1 (MQ=255)`  
                                                                           `aaCTTGACGTAATCGACAAGCAATTCCAGAAGCGAATCAACGGTGAGTGGGTCTCAACGGATTTTAAGGATATGCCAGTCAACGAGAACACTGGTGAGTTCAAGCATAGCGATATGGTTAACTACGCCAATAAGAAGCTCGCTGAGATTGACAGTATGGACATTCCAGACGGTGCCAAGGATGCTATGAAGTTGAAGTACCTTCAAGCGGACTCTAAGGACGGAGTATTCCGTACAGCCATCGGAACCATGGTCACTGACGCTGGTCAAGAGTGGTCTGCCGCTGTGactgtctcttatac                                                                                                                                                                                                                                  >  1:388053/1‑288 (MQ=255)`  
                                                                           `aaCTTGACGTAATCGACAAGCAATTCCAGAAGCGAATCAACGGTGAGTGGGTCTCAACGGATTTTAAGGATATGCCAGTCAACGAGAACACTGGTGAGTTCAAGCATAGCGATATGGTTAACTACGCCAATAAGAAGCTCGCTGAGATTGACAGTATGGACATTCCAGACGGTGCCAAGGATGCTATGAAGTTGAAGTACCTTCAAGCGGACTCTAAGGACGGAGTATTCCGTACAGCCATCGGAACCATGGTCACTGACGCTGGTCAAGAGTGGTCTGCCGCTGTGATTAACGGTAAGtt                                                                                                                                                                                                                                  >  1:509803/1‑301 (MQ=255)`  
                                                                                    `tAATCGACAAGCAATTCCAGAAGCGAATCAACGGTGAGTGGGTCTCAACGGATTTTAAGGATATGCCAGTCAACGAGAACACTGGTGAGTTCAAGCATAGCGATATGGTTAACTACGCCAATAAGAAGCTCGCTGAGATTGACAGTATGGACATTCCAGACGGTGCCAAGGATGCTATGAAGTTGAAGTACCTTCAAGCGGACTCTAAGGACGGAGTATTCCGTACAGCCATCGGAACCATGGTCACTGACGCTGGTCAAGAGTGGTCTGCCGCTGTGATTAACGGTAAGTTACCAgaacg                                                                                                                                                                                                                         >  1:553947/1‑301 (MQ=255)`  
                                                                                      `aTCGACAAGCAATTCCAGAAGCGAATCAACGGTGAGTGGGTCTCAACGGATTTTAAGGATATGCCAGTCAACGAGAACACTGGTGAGTTCAAGTATAGCGATATGGTTAACTACGCCAATAAGAAGCTCGCTGAGATTGACAGTATGGACATTCCAGACGGTGCCAAGGATGCTATGAAGTTGAAGTACCTTCAAGCGGACTCTAAGGACGGAGCATTCCGTACAGCCATCGGAACCATGGTCACTGACGCTGGTCAAGAGTGGTCGGCCGCTGTGATTAACGGTAAGTTACCAgaacgaa                                                                                                                                                                                                                       >  1:410416/1‑301 (MQ=255)`  
                                                                                          `ataggtgtataagagacagAATCAACGGTGAGTGGGTCTCAACGGATTTTAAGGATATGCCAGTCAACGAGAACACTGGTGAGTTCAAGCATAGCGATATGGTTAACTACGCCAATAAGAAGCTCGCTGAGATTGACAGTATGGACATTACAGACGGTGCCAAGGATGCTATGAAGTTGAAGTACCTTCAAGCGGACTCTAAGGACGGAGTATTCCGTACAGCAATCGGAACCATGGTCACTGACGCTGGTCAAGAGTGGTCTGCCGCTGTGATTAACGGTAAGTTACCAGAACGAAaccc                                                                                                                                                                                                                   <  1:380006/283‑1 (MQ=255)`  
                                                                                               `cAATTCCAGAAGCGAAGCAACGGTGAGTGGGTCTCAACGGATTTTAAGGATATGCCAGTCAACGAGAACACTGGTGAGTTCAAGCATAGCGATATGGTTAACTACGCCAATAAGAAGCTCGCTGAGATTGACAGTATGGACATTCCAGACGGTGCCAAGGATGCTATGAAGTTGAAGTACCTTCAAGCGGACTCTAAGGACGGAGTATTCCGTACAGCCATCGGAACCATGGTCACTAACGCTGGTCAAGAGTGGTCTGCCGCTGTGATTAACGGTAAGTTACCAGAACGAACCCCAGCTa                                                                                                                                                                                                              <  1:503208/301‑1 (MQ=255)`  
                                                                                                  `agccAGAAGCGAATCAACGGTGAGTGGGTCTCAACGGATTTTAAGGATATGCCAGTCAACGAGAACACTGGTGAGTTCAAGCAGAGCGATATGGTTAACTACGCCAATAAGAAGCTCGCTGAGATTGACAGTATGGACATTCCAGACGGTGCCAAGGATGCTATGAAGTTGAAGTACCTTCAAGCGGACTCTAAGGACGGAGTATTCCGTACAGCCATCGGAACCATGGTCACTGACGCTGGTCAAGAGTGGTCTGCCGCTGTGATTAACGGTAAGTTACCAGAACGAACCCCAGCTATgg                                                                                                                                                                                                           <  1:340621/299‑1 (MQ=255)`  
                                                                                                   `tCCAGAAGCGAATCAACGGTGAGTGGGTCTCAACGGATTGTAAGGAGAGGCCAGTAAACGAGAACACTGGTGAGTTCAAGCATAGCGATATGGTTAACTACGCCAATAAGAAGCTCGCTGAGATTGACAGTATGGACATTCCAGACGGTGCCAAGGATGCTATGAAGTTGAAGTACCTTCAAGCGGACTCTAAGGACGGAGTATTCCGTACAGCCATCGGAACCATGGTCACTGACGCTGGTCAAGAGTGGTCTGCCGCTGTGATTAACGGTAAGTTACCAGAACGAACCCCAGCTATGGa                                                                                                                                                                                                          <  1:40423/301‑1 (MQ=255)`  
                                                                                                     `cAGAAGAGAATCAACGGTGAGTGGGTCTCAACGGATTTTAAGGATATGCCAGTCAACGAGAACACTGGTGAGTTCAAGCATAGCGATATGGTTAACTACGCCAATAAGAAGCTCGCTGAGATTGACAGTATGGACATTCCAGACGGTGCCAAGGATGCTATGAAGTTGAAGTACCTTCAAGCGGACTCTAAGGGCGGAGCATTCCGTACAGCCATCGGAACCATGGTCACTGACGCTGGTCAAGAGTGGTCTGCCGCTGTGATTAACGGTAAGTTACCAGAACGAACCCCAGCTATGGATg                                                                                                                                                                                                        <  1:234042/301‑1 (MQ=255)`  
                                                                                                      `aGAAGCGAATCAACGGTGAGTGGGTCTCAACGGATTTTAAGGATATGCCAGTCAACGAGAACACTGGTGAGTTCAAGCATAGCGATATGGTTAACTACGCCAATAAGAAGCTCGCTGAGATTGACAGTATGGACATTCCAGACGGTGCCAAGGATGCTATGAAGTTGAAGTACCTTCAAGCGGACTCTAAGTACGGAGTATTCCGTACAGCCATCGGAACCATGGTCACTGACGCTGGTCAAGAGTGGTCTGCCGCTGTGATTAACGGTAAGTTACCAGAACGAACTCCAGATATGGATGc                                                                                                                                                                                                       <  1:32901/301‑1 (MQ=255)`  
                                                                                                        `atgtgtataagagacaggTGGGTCTCAACGGATTTTAAGGATATGCCAGTCAACGAGAACACTGGTGAGTTCAAGCATAGCGATATGGTTAACTACGCCAATAAGAAGCTCGCTGAGATTGACAGTATGGACATTCCAGACGGTGCCAAGGATGCTATGAAGTTGAAGTACCTTCAAGCGGACTCTAAGGACGGAGTATTCCGTACAGCCATCGGAACCATGGTCACTGACGCTGGTCAAGAGTGGTCTGCCGCTGTGATTAACGGTAAGTTACCAGAACGAACCCCAGCTATGGATGctc                                                                                                                                                                                                     <  1:585555/284‑1 (MQ=255)`  
                                                                                                          `gCGAATCAACGGTGAGTGGGTCTCAACGGATTTTAAGGATATGCCAGTCAACGAGAACACTGGTGAGTTCAAGCATAGCGATATGGTTAACTACGCCAATAAGAAGCTCGCTGAGATTGACAGTATGGACATTCCAGACGTTGCCAAGGATGCTATGAAGTTGAAGTACCTTCAAGCGGACTCTAAGGACGGAGTATTCCGTACAGCCATCGGAACCATGGTCACTGACGCTGGTCAAGAGTGGTCTGCCGCTGTGATTAACGGTAAGTTACCAGAACGAACCCCAGCTATGGATGCTCTg                                                                                                                                                                                                   >  1:323637/1‑301 (MQ=255)`  
                                                                                                           `cGAATCAACGGTGAGTGGGTCTCAACGGATTTTAAGGATATGCCAGTCAACGAGAACACTGGTGAGTTCAAGCATAGCGATATGGTTAACTACGCCAATAAGAAGCTCGCTGAGATTGACAGTATGGACATTCCAGACGGTGCCAAGGATGCTATGAAGTTGAAGTACCTTCAAGCGGACTCTAAGGACGGAGTATTCCGTACAGCCATCGGAACCATGGTCACTGACGCTGGTCAAGAGTGGTCTGCCGCTGTGATTAACGGTAAGTTACCAGAACGAACCCCAGATATGGATGCTCTgc                                                                                                                                                                                                  >  1:516496/1‑301 (MQ=255)`  
                                                                                                             `aaTCAACGGTGAGTGGGTCTCAACGGATTTTAAGGATATGCCAGTCAACGAGAACACTGGTGAGTTCAAGCATAGCGATATGGTTAACTACGCCAATAAGAAGCTCGCTGAGATTGACAGTATGTACATTCTAGACGGTGCCAAGGATGCCATGAAGCTGAAGTACCTTCAAGCGGACTCTAAGGACCGAGTATTCCGTAAAGCCATCAGAACCATTGTGAATGACGCTGGGTAAGACTGGTCTGCCGATGTGATTAACGGTAAGTTACCAGAACCAA‑CCCAGACCTGGATGCTCTGCGCa                                                                                                                                                                                               >  1:134084/1‑301 (MQ=255)`  
                                                                                                             `aaTCAACGGTGAGTGGGTCTCAACGGATTTTAAGGATATGCCAGTCAACGAGAAAACTGGGTAGTTCAAGCATAGCGATATGGTTAACTACGCCAATAAGAAGCTCGCTGAGATTGACAGTATGGACATTCCAGACGGTGCCAAGGATGCTATGAAGTTGAAGTACCTTCAAGCGGACTCTAAGGACGGAGCATTCCGTACAGCCATCGGAACCATGGTCACTGACGCTGGTCAAGAGTGGTCTGCCGCTGTGATTAACGGTAAGTTACCAGAACGAACCCCAGCTATGGATGCTCTgcgc                                                                                                                                                                                                <  1:53496/301‑1 (MQ=255)`  
                                                                                                               `tCAATGGTGAGTGGGTCTCAACGGATTTTAAGGATATGCCAGTCAACGAGAACACTGGTGAGTTCAAGTATAGCGATATGGTTAACTACGCCAATAAGAAGCTCGCTGAGATTGACAGTATGGACATTCCAGACGGTGCCAAGGATGCTATGAAGTTGAAGTACCTTCAAGCGGACTCTAAGGACGGCGCATTCCGTACAGCCATCGGAACCATGGTCACTGACGCTGGTCAAGAGTGGTCTGCCGCTGTGATTAACGGTAAGTTACCAGACCAAACCCCAGCTATGGATGCTCTGCGCAg                                                                                                                                                                                              >  1:82012/1‑301 (MQ=255)`  
                                                                                                               `tCAACGGTGAGTGGGTCTCAACGGATTTTAAGGAGATGCCAGTCAACGAGAAAACTGGTGAGTTCAAGCATAGCGATATGGTTAACTACGCCAATAAGAAGCTCGCTGAGATTGACAGTATGGACATTCCAGACGGTGCCAAGGATGCTATGAAGTTGAAGTACCTTCAAGCGGACTCTAAGGACGTAGTATTCCGTACAGCCATCGGAACCATGGTCACTGACGCTGGTCAAGAGTGGTCTGCCGCTGTGATTAACGGTAAGTTACCAGAACGAACCCCAGCTATGGATGCTCTGCGCAg                                                                                                                                                                                              <  1:43634/301‑1 (MQ=255)`  
                                                                                                                 `aaCGGTGAGTGGGTCTCAACGGATTTTAAGGATATGCCAGTCAACGAGAACACTGGTGAGTTCAAGTATATCGATATGGTTAACTACGCCAATAAGAAGCTCGCTGAGATTGACAGTATGGACATTCCAGACGGTGCCAAGGATGCTATGAAGTTGAAGTACCTTCAAGCGGTCTCTAAGGACGGAGCATTCCGTACAGCCATCGGAACCATGGTCACTGACGCTGGTCAAGAGTGGTCTGCCGCTGTGATTAACGGTAAGTTACCAGAACGAACCCCAGCTATGGATGCTCTGCGCAGaa                                                                                                                                                                                            <  1:145286/301‑1 (MQ=255)`  
                                                                                                                            `ggTCTCAACGGATTTTAAGGATATGCCAGTCAACGAGAACACTGGTGAGTTCAAGCATAGCGATATGGTTAACTACGCCAATAAGAAGCTCGCTGAGATTGACAGTATGGACATTCCAGACGGTGCCAAGGATGCTATGAAGTTGAAGTACCTTCAAGCGGACTCTAAGGACGGAGTATTCCGTACAGCCATCGGAACCATGGTCACTGACGCTGGTCAAGAGTGGTCTGCCGCTGTGATTAACGGTAAGTTACCAGAACGAACCCCAGCTATGGATGCTCTGCGCAGAATCCGCAATGCt                                                                                                                                                                                 >  1:368600/1‑301 (MQ=255)`  
                                                                                                                            `ggTCTCAACGGATTTTAAGGATATGCCAGTCAACGAGAACACTGGTGAGTTCAAGCATAGCGATATGGTTAACTACGCCAATAAGAAGCTCGCTGAGATTGACAGTATGGACATTCCAGACGGTGCCAAGGATGCTATGAAGTTGAAGTACCTTCAAGCGGACTCTAAGGACGGAGTATTCCGTACAGCCATCGGAACCATGGTCACTGACGCTGGTCAAGAGTGGTCTGCCGCTGTGATTAACGGTAAGTTACCAGAAAGAACCCCAGCTAGGGATGATCTGCGCAGAATCCGCAATGCt                                                                                                                                                                                 >  1:257663/1‑301 (MQ=255)`  
                                                                                                                                 `cAACGGATTTTAAGGATATGCCAGTCAACGAGAACACTGGTGAGTTCAAGTATAGCGATATGGTTAACTACGCCAATAAGAAGCTCGCTGAGATTGACAGTATGGACATTCCAGACGGTGCCAAGGATGCTATGAAGTTGAAGTACCTTCAAGCGGACTCTAAGGACGGAGCATTCCGTACAGCCATCGGAACCATGGTCACTGACGCTGGTCAAGAGTGGTCTGCCGCTGTGATTAACGGTAAGTTACCAGAACGAACCCCAGCTATGGATGCTCTGCGCAGAATCCGCAATGCTGAccc                                                                                                                                                                            >  1:292727/1‑301 (MQ=255)`  
                                                                                                                                 `cAACGGATTTTAAGGATATGCCAGTCAACGAGAACACTGGTGAGTTCAAGCATAGCGATATGGTTAACTACGCCAATAAGAAGCTCGCTGAGATTGACAGTATGGACATTCCAGACGGTGCCAAGGATGCTATGAAGTTGAAGTACCTTCAAGCGGACTCTAAGGACGGAGTATTCCGTACAGCCATCGGAACCATGGTCACTGACGCTGGTCAAGAGTGGTCTGCCGCTGTGATTAACGGTAAGTTACCAGAACGAACCCCAGCTATGGATGCTCTGCGCAGAATCCGCAATGCTGAccc                                                                                                                                                                            >  1:375228/1‑301 (MQ=255)`  
                                                                                                                                      `gATTTTAAGGATATGCCAGTCAACGAGAACACTGGTGAGTTCAAGCATAGCGATATGGTTAACTACGCCAATAAGAAGCTCGATGAGATTGACAGTATGGACATTCCAGACGGTGCCAAGGATGCTATGAAGTTGAAGTACCTTCAAGCGGACTCTAAGGACGGAGTATTCCGTACAGCCATCGGAACCATGGTCACTGACGCTGGTCAAGAGTGGTCTGCCGCTGTGATTAACGGTAAGTTACCAGAACGAACCCCAGCTATGGATGCTCTGCGCAGAATCCGCAATGCTGACCCTCAGt                                                                                                                                                                       <  1:609684/301‑1 (MQ=255)`  
                                                                                                                                      `aaatttAAGGAAATGCCAGTCAACGAGAACACTGGTGAGTTCAAGAATAGCGATATGGTTAACTACGCCAATAAGAAGCTCGCTGAGATTGACAGTATGGACATTCCAGACGGTGCCAAGGATGCTATGAAGTTGAAGTACCTTCAAGCGGACTCTAAGGACGGAGTATTCCGTACAGCCATCGGAACCATGGTCACTGACGATGGTCAAGAGTGGTCTGCCGCTGTGATTAATGGTAAGTTACCAGAACGAACCCCAGCTATGGATGCTCTGCGCAGAATCCGCAATGCTGACCCTCAGt                                                                                                                                                                       <  1:139979/298‑1 (MQ=255)`  
                                                                                                                                              `ggATATGCCAGTCAACGAGAACACTGGTGAGTTCAAGCATAGCGATATGGTTAACTACGCCAATAAGAAGCTCGCTGAGATTGACAGTATGGACATTCCAGACGGTGCCAAGGATGCTATGAAGTTGAAGTACCTTCAAGCGGACTCTAAGGACGGAGTATTCCGTACAGCCATCGGAACCATGGTCACTGACGCTGGTCAAGAGTGGTCTGCCGCTGTGATTAACGGTAAGTTACCAGAACGAACCCCAGCTATGGATGCTCTGCGCAGAATCCGCAATGCTGACCCTCAGTTGATtgct                                                                                                                                                               >  1:310899/1‑301 (MQ=255)`  
                                                                                                                                                         `tgtagaagagacagggTGAGTACAAGCATAGCGATATGGTTAAATACGCCAATAAGAAGCTCGCTGAGATTGACAGTATGGAAATTCCAGAAGGTGCCAAGGATGCTATGAAGTTGAAGTACCTTCAAGCGGACTCTAAGGACGGAGTATTCCGTACAGCCATCGGAAACATGGTCACTGACGCTGGTCAAGAGTGGTCTGCCGCTGTGATTAACGGTAAGTTACCAGAACGAACCCCAGCTATGGATGCTCTGCGCAGAATCCGCAATGCTGACCCTCAGTTGATTGCTGCGCTATAccc                                                                                                                                                    <  1:57843/287‑1 (MQ=255)`  
                                                                                                                                                           `aaCGAGAACACTGGTGAGTTCAAGCATAGCGATATGGTTAACTACGCCAATAAGAAGCTCGCTGAGATTGACAGTATGGACATTCCAGACGGTGCCAAGGATGCTATGAAGTTGAAGTACCTTCAAGCGGACTCTAAGGACGGAGTATTCCGTACAGCCATCGGAACCATGGTCACTGACGCTGGTCAAGAGTGGTCTGCCGATGTGATTAACGGTAAGTTACCAGAACGAACCCCAGCTATGGATGCTCTGCGCAGAATCCGCAATGCTGACCCTCAGTTGATTGCTGCGCTATACCCAg                                                                                                                                                  >  1:169556/1‑301 (MQ=255)`  
                                                                                                                                                             `cGAGAACACTGGTGAGTTCAAGTATAGCGATATGGTTAACTACGCCAATAAGAAGCTCGCTGAGATTGACAGTATGGACATTCCAGACGGTGCCAAGGATGCTATGAAGTTGAAGTACCTTCAAGCGGACTCTAAGGACGGGGCATTCCGTACAGCCATCGGAACCATGGTCACTGACGCTGGTCAAGAGTGGTCTTCCGCTGTGATTAACGGTAAGTTACCAGAACGAACCCCAGCTATGGATGCTCTGCGCAGAATCCGCAATGCTGACCCTCAGTTGATTGCTGCGCTATACCCAGAc                                                                                                                                                <  1:522618/301‑1 (MQ=255)`  
                                                                                                                                                             `agagaACACTGGTGAGTTCAAGCATAGCGATATGGTTAACGAAGCCAATAAGAAGCTCGCTGAGATTGACAGTATGGACAATCCAGACGGGGCCAAGGATGCTATGAAGTTGAAGTACCTTCAAGCGGACTCTAAGGACGGAGCATTCCGTACAGCCATCGGAAACATGGTCACTGACGCTGGTCAAGAGTGGTCTGCCGCTGTGATTAACGGTAAGTTAACAGAACGAACCCCAGCTATGGATGCTCTGCGCAGAATCCGCAATGCTGACCCTCAGTTGATTGCTGCGCTATACCCAGAc                                                                                                                                                <  1:287597/300‑1 (MQ=255)`  
                                                                                                                                                              `gagaACACTGGTGAGTTCAAGCATAGCGATATGGTTAACTACGCCAATAAGAAGCTCGCTGAGATTGACAGTATGGACATTCCAGACGGTGCCAAGGATGCTATGAAGTTGAAGTACCTTCAAGCGGACTCTAAGGACGGAGTATTCCGTACAGCCATCGGAACCATGGTCACTGACGCTGGTCAAGAGTGGTCTGCCGCTGTGATTAACGGTAAGTTACCAGAACGAACCCCAGCTATGGATGCTCTGCGCAGAATCCGCAATGCTGACCCTCAGTTGATTGCTGCGCTATACCCAGAcc                                                                                                                                               <  1:431845/301‑1 (MQ=255)`  
                                                                                                                                                               `acgacacTGGTTCGTACAATCAGAGCGATATGGTTAACTACGCCAATAAGAAGCTCGCTGAGATTGACAGTATGGACATACCAGACGTTGCCAAGGATGCTATGAAGTTGAAGTAGCTTCAAGCGCACTCTAAGGGCGGAGCATTCCGTACAGCAATCTGAACCCTGGTGACTTACTCTGGTCAAGTGTGTTCTCCCGCTGTGATTAACGGTAAGTCACCAGAACGATCCCCAGCTATGGATGCTCTGCGCAGAATCCGCAATGCTGACCCTCAGTTGATTGCTGCGCTATACCCAGACCa                                                                                                                                              <  1:474436/298‑1 (MQ=255)`  
                                                                                                                                                                   `cacTGGTGAGTTCAAGCATAGCGATATGGTTAACTACGCCAATAAGAAGCTCGCTGAGATTGACAGTATGGACATTCCAGACGGTGCCAAGGATGCTATGAAGTTGAAGTACCTTCAAGCGGACTCTAAGGACGGAGTATTCCGTACAGCCGTCGGAACCATGGTCACTGACGCTGGTCAAGAGTGGTCTGCCGCTGTGATTAACGGTAAGTTACCAGAACGAACCCCAGCTATGGATGCTCTGCGCAGAATCCGCAATGCTGACCCTCCGTTGATTGCTGCGCTATACCCAGACCAAGCt                                                                                                                                          >  1:112165/1‑301 (MQ=255)`  
                                                                                                                                                                    `agagacaggTTCAAGCATAGCGATATTGTTAACTACGCCAATAAGAAGCTCGCTGAGATTGACAGTATGGACATTCCAGACGGTGCCAAGGATGCTATGAAGTTGAAGTACCTTCAAGCGGACTCTAAGGACGGAGTATTCCGTACAGCCATCGGAACCATGGTCACTGACGCTGGTCAAGAGTGGTCTGCCGCTGTGATTAACGGTAAGTTACCAGAACGAACCCCAGCTATGGATGCTCTGCGCAGAATCCGCAATGCTGACCCTCAGTTGATTGCTGCGCTATACCCAGACCAAGCTg                                                                                                                                         <  1:441164/293‑1 (MQ=255)`  
                                                                                                                                                                       `ggTGAGTTCAAGTATAGCGATATGGTTAACTACGCCAATAAGAAGCTCGCTGAGATTGACAGTATGGACATTCCAGACGGTGCCAAGGATGCTATGAAGTTGAAGTACCTTCAAGCGGACTCTAAGGACGGAGCATTCCGTACAGCCATCGGAACCATGGTCACTGACGCTGGTCAAGAGTGGTCTGCCGCTGTGATTAACGGTAAGTTACCAGAACGAACCCCAGCTATGGATGCTATGCGCAGAATCCGCAATGCTGACCCTCAGTTGATTGCTGCGCAATACCCAGACAAAGCTGAGc                                                                                                                                      >  1:637157/1‑301 (MQ=255)`  
                                                                                                                                                                       `ggTGAGTTCAAGCATAGCGATATGGTTAACTACGCCAATAAGAAGCTCGCTGAGATTGACAGTATGGACATTCCAGACGGTGCCAAGGATGCTATGAAGTTGAAGTACCTTCAAGCGGACTCTAAGGGCGGAGCATTCCGTACAGCCATCGGAACCATGGTCACTGACGCTGGTCAAGAGTGGTCTGCCGCTGTGATTAACGGTAAGTTACCAGAACGAACCCCAGCTATGGATGCTCTGCGCAGAATCCGCAATGCTGACCCTCAGTTGATTGCTGCGCTATACCCAGACCAAGCTGAGc                                                                                                                                      <  1:129400/301‑1 (MQ=255)`  
                                                                                                                                                                       `ggTGAGTTCAAGCATAGCGATATGGTTAACTACGCCAATAAGAAGCTCGCTGAGATTGACAGTATGGACATTCCAGACGGTGCCAAGGATGCTATGAAGTTGAAGTACCTTCAAGCGGACTCTAAGGACGGAGTATTCCGTACAGCCATCGGAACCATGGTCACTGACGCTGGTCAAGAGTGGTCTGCCGCTGTGATTAACGGTAAGTTACCAGAACGAACCCCAGCTATGGATGCTCTGCGCAGAATCCGCAATGCTGACCCTCAGTTGATTGCTGCGCTATACCCAGAcctgtctctta                                                                                                                                      >  1:357410/1‑292 (MQ=255)`  
                                                                                                                                                                            `gTTCAAGCATAGCGATATGGTTAACTACGCCAATAAGAAGCTCGCTGAGATTGACAGTATGGACATTCCAGACGGTGCCAAGGATGCTATGACGTTGAAGTACCTTCAAGCGGACTCTAAGGACGGCGTATTCCGTACAGCCATCGGAACCATGGTCACTGACGCTGGTCAAGAGTGGTCTGCCGCTGTGATTAACGGTAAGTTACCAGAACGAACCCCAGCTATGGATGCTCTGCGCAGAATCCGCAATGCTGACCCTCAGTTGATTGCTGCGCTATAcccctgtctcttatacacatct                                                                                                                                 >  1:100532/1‑282 (MQ=255)`  
                                                                                                                                                                            `gTTCAAGCATAGCGATATGGTTAACTACGCCAATAAGAAGCTCGCTGAGATTGACAGTATGGACATTCCAGACGGTGCCAAGGATGCTATGAAGTTGAAGTACCTTCAAGCGGACTCTAAGGACGGAGTATTCCGTACAGCCATCGGAACCATGGTCACTGACGCTGGTCAAGAGTGGTCTGCCGCTGTGATTAACGGTAAGTTACCAGAACGAACCCCAGCTATGGATGCTCTGCGCAGAATCCGCAATGCTGACCCTCAGTTGATTGCTGCGCTATACCCAGACCAAGCTGAGCTATTc                                                                                                                                 <  1:227314/301‑1 (MQ=255)`  
                                                                                                                                                                            `attCAAGCATAGCGATATGGTTAACTACGCCAATAAGAAGCTCGCTGAGATTGACAGTATGGACATTCCAGACGGTGCCAAGGATGCTATGAAGTTGAAGTACCTTCAAGTGGACTCTAAGGACGGAGTATTCCGTACAGCCATCGGAACCATGGTCACTGACGCTGGTCAAGAGTGGTCTGCCGCCGTGATTAACGGTAAGTTACCAGAACGAACCCCAGCTATGGATGCTCTGCGCAGAATCCGCAATGCTGACCCTCAGTTGATTGCTGCGCTATACCCAGACCAAGCTGAGCTATTc                                                                                                                                 <  1:253100/300‑1 (MQ=255)`  
                                                                                                                                                                               `cAAGCATAGCGATATGGTTAACTACGCCAATAAGAAGCTCGCTGAGATTGACAGTATGGACATTCCAGACGGTGCCAAGGATGCTATGAAGTTGAAGTACCTTCAAGCGGACTCTAAGGACGGAGCATTCCGTACAGCCATCGGAACCATGGTCACTGACGCTGGTCAAGAGTGGTCTGCCGCTGTGATTAACGGTAAGTTACCAGAACGAACCCCAGCTATGGATGCTCTGCGCAGAATCCGCAATGCTGACCCTCAGTTGATTGCTGCGCTATACCCAGACCAAGCTGAGCTATTCCTg                                                                                                                              >  1:659162/1‑301 (MQ=255)`  
                                                                                                                                                                                   `cATAGCTATATGGTTAACTACGCCAATAAGAAGCTCGCTGAGATTGACAGTATGGACATTCCAGACGGTGCCAAGGATGCTATGAAGTTGAAGTACCTTCAAGCGGACTCTAAGGACGGAGCATTCCGTACAGCCATCGGAACCATGGTCACTGACGCTGGTCAAGAGTGGTCTGCCGCTGTGATTAACGGTAAGTTACCAGAACGAACCCCAGCTATGGATGCTCTGCGCAGAATCCGCAATGCTGACCCTCAGTTGATTGCTGCGCTATACCCAGACCAAGCTGAGCTATTCCTGACGa                                                                                                                          <  1:316218/301‑1 (MQ=255)`  
                                                                                                                                                                                     `tAGCGATATGGTTAACTACGCCAATAAGAAGCTCGCTGAGATTGACAGTATGGACATTCCAGACGGTGCCAAGGATGCTATGAAGTTGAAGTACCTTCAAGCGGACTCTAAGGACGGAGTATTCCGTACAGCCATCGGAACCATGGTCACTGACGCTGGTCAAGAGTGGTCTGCCGCTGTGATTAACGGTAAGTTACCAGAACGAACCCCAGCTATGGATGCTCTGCGCAGAATCCGCAATGCTGACCCTCAGTTGATTGCTGCGATATACCCAGACAAAGCTGAGCTATTCCTGACGATg                                                                                                                        >  1:602770/1‑301 (MQ=255)`  
                                                                                                                                                                                       `gCGATATGGTTAACTACGCCAATAAGAAGCTCGCTGAGATTGACAGTATGGACATTCCAGACGGTGCCAAGGATGCTATGAAGTTGAAGTACCTTCAAGCGGACTCTAAGGACGGAGTATTCCGTACAGCCATCGGAACCATGGTCACTGACGCTGGTCAAGAGTGGTCTGCCGCTGTGATTAACGGTAAGTTACCAGAACGAACCCCAGCTATGGATGCTCTGCGCAGAATCCGCAATGCTGACCCTCAGTTGATTGCTGCGCTATACCCAGACCAAGCTGAGCTATTCCTgtctcttat                                                                                                                      >  1:572625/1‑293 (MQ=255)`  
                                                                                                                                                                                          `atatGGTTAACTACGCCAATAAGAAGCTCGCTGAGATTGACAGTATGGACATTCCAGACGGTGCCAAGGATGCTATGAAGTTGAAGTACCTTCAAGCGGACTCTAAGGACGGAGTATTCCGTACAGCCATCGGAACCATGGTCACTGACGCTGGTCAAGAGTGGTCTGCCGCTGTGATTAACGGTAAGTTACCAGAACGAACCCCAGCTTTGGATGCTCTTCGCAGAATCAGCAATGCTGACCCTCAGTTGATTGCTGCGCTATACCCAGACCAAGCTGAGCTATTCCTGACGATGGACat                                                                                                                   >  1:270008/1‑301 (MQ=255)`  
                                                                                                                                                                                          `agagacagaaCTACGCCAATAAGAAGCGCGCTGAGATTGACAGTATGGACATTCCAGACGGTGCCAAGGATGCTATGAAGTTGAAGTACCGTCAAGCGGACTCTAAGGACGGAGTATTCCGTACAGCCATCGGAACCATGGTCACTGACGCTGGTCAAGAGTGGTCTGCCGCTGTGATTAACGGTAAGTTACCAGAACGAACCCCAGCTATGGATGCTCTGCGCAGAATCCGCCATGCTGACCCTCAGTTGATTGCTGCGCTATACCCAGACCAAGCTGAGCTATTCCTGACGATGGACat                                                                                                                   <  1:643131/293‑1 (MQ=255)`  
                                                                                                                                                                                             `tGGTTAACTACGCCAATAAGAAGCTCGCTGAGATTGACAGTATGGACATTCCAGACGGTGCCAAGGATGCTATGAAGTTGAAGTACCTTCAAGCGGACTCTAAGGACGGAGTATTCCGTACAGCCATCGGAACCATGGTCACTGACGCTGGTCAAGAGTGGTCTGCCGCTGTGATTAACGGTAAGTTACCAGAACGAACCCCAGCTATGGATGCTCTGCGCAGAATCCGCAATGCTGACCCTCAGTTGATTGCTGCGCTATACCCAGACCAAGCTGAGCTATTCCTGACGATGGACatgat                                                                                                                >  1:175926/1‑301 (MQ=255)`  
                                                                                                                                                                                             `tGGTTAACTACGCCAATAAGAAGCTCGCTGAGATTGACAGTATGGACATTCCAGACGGTGCCAAGGATGCTATGAAGTTGAAGTACCTTCAAGCGGACTCTAAGGACGGAGTATTCCGTACAGCCATCGGAACCATGATCACTGACGCTGGTCAAGAGTGGTCTGCCGCTGTGATTAACGGTAAGTTACCAGAACGAACCCCAGCTATGGATGCTCTGCGCAGAATCCGCAATGCTGACCCTCAGTTGATTGCTGCGCTCTACCCAGACCAAGCTGAGCTATTCCTGAcctgtctcttata                                                                                                                >  1:299008/1‑289 (MQ=255)`  
                                                                                                                                                                                             `tGGTTAACTACGCCAATAAGAAGCTCGCTGAGATTGACAGTATGGACATTCCAGACGGTGCCAAGGATGCTATGAAGTTGAAGTACCTTCAAGCGGACTCTAAGGACGGAGCATTCCGTACAGCCATCGGAACCATGGTCACTGACGCTGGTCAAGAGTGGTCTGCCGCTGTGATTAACGGTAAGTTACCAGAACGAACCCCAGCTATGGATGCTCTGCGCAGAATCCGCAATGCTGACCCTCAGTTGATTGCTGCGCTATACCCAGACCAAGCTGAGCTATTCCTGACGATGGACatgat                                                                                                                <  1:93285/301‑1 (MQ=255)`  
                                                                                                                                                                                               `gTTAACTACGCCAATAAGAAGCTCGCTGAGATTGACAGTATGGACATTCCAGACGGTGCCAAGGATGCTATGAAGTTGAAGTACCTTCAAGCGGACTCTAAGGACGGAGTAGTCCGTACAGCCATCGGAACCATGGTCACTGACGCTGGTCAAGAGTGGTCTGCCGCTGTGATTAACGGTAAGTTACCAGAACGAACCCCAGCTATGGATGCTCTGCGCAGAATCCGCAATGCTGACCCTCAGTTGATTGCTGCGCTATACCCAGACCAAGCTGAGCTATTCCTGACGATGGACATGATgg                                                                                                              <  1:211868/301‑1 (MQ=255)`  
                                                                                                                                                                                                 `tAACTACGCCAATAAGAAGCTCGCTGAGATTGACAGTATGGACATTCCAGCCGGTGCCAAGGATGCCATGAAGTTGAAGTACCTTCAAGAGGACTCTAAGGACGGAGTATTCCGTACAGCCATCGGAACCATTGTCACTGACGCTGGTCAAGAGTGGTCTGCCGCTGTGATTTACTGTTAGTTACCAGACCGAATCCCAGCTCTGGATGCTCTGCGCCGAATCCGCCATTCTGACCCTCAGTTGATTGCTGCGCTATACCCACACCAAGCTGAGCTATTTCTCACGATTGAGACGTTGGAc                                                                                                            >  1:630236/1‑301 (MQ=255)`  
                                                                                                                                                                                                      `aCGCCAATAAGAAGCGCGCTGAGATTGACATTATGGACATTCCAGACGGTGCCAATGATGCTATGAAGTTGAAGTACCTTCAAGCGGACTCTAAGGCCGGCGCATTCCGTACAGCCATCGGAACCATGGTCACTGACGCTGGTCAAGCGTGGTCTGCCGCTGTGATTAACGGTAAGTCACCAGAACGCACCCCAGCTATGGATGCTCTGCGCAGAATCCGCAATGCTGACCCTCAGTTGATTGCTGCGCTATACCCAGACCTAGCTGAGCTATTCCTGACGATGGACATGATGGACAAGCa                                                                                                       <  1:455026/301‑1 (MQ=255)`  
                                                                                                                                                                                                                `gaagCTCGCTGAGATTGACAGTATGGACATTCCAGACGGTGCCAAGGATGCTATGAAGTTGAAGTACCTTCAAGCGGACTCTAAGGACGGAGTATTCAGTACAGCCATCGGAACCATGGTCACTGACGCTGGTCAAGAGTGGTCTGCCGCTGTGATTAACGGTAAGTTACCAGAACGAACCCCAGCTATGGATGCTCTGCGCAGAATCCGCAATGCTGACCCTCAGTTGATTGCTGCGCTATACCCAGACCAAGCTGAGCTATTCCTGACGATGGACATGATGGACAAGCAGGGTATTGAc                                                                                             <  1:152054/301‑1 (MQ=255)`  
                                                                                                                                                                                                                 `aagCTCGCTGAGATTGACAGTATGGACATTCCAGACGGTGCCAAGGATGCTATGAAGTTGAAGTACCTTCAAGCGGACTCTAAGGACGGAGCATTCCGTACAGCCATCGGAACCATGGTCACTGACGCTGGTCAAGAGTGGTCTGGCGCTGTGATTAACGGTAAGTTACCAGAACGAACCCCAGCTATGGATGCTCTGCGCAGAATCCGCAATGCTGACCCTCAGTTGATTGCTGCGCTATACCCAGACCAAGCTGAGCTATTCCTGACGATGGACATGATGGACAAGCAGGGTATTGAcc                                                                                            <  1:334945/301‑1 (MQ=255)`  
                                                                                                                                                                                                                 `aagCTCGCTGAGATTGACAGTATGGACATTCCAGACGGAGCCAAGGATGATATGAAGTTGAAGGACCGTCAAGCGGACTCTAAGGACGGAGTATGCCGTACAGCCAGCGGAAACATGGTCACTGACGCGGGTCAAGAGTGGTCTGCCGCTGTGATAAACGGTAAGTTACCAGAACGAACCCCAGCGATGGATGCTCTGCGCAGAATCCGCAATGCTGACCCTCAGTTGATTGCTACGCTATACCCAGACCAAGCTGAGCTATTCCTGACGATGGACATGATGGACAAGCAGGGTATTGAcc                                                                                            <  1:667103/301‑1 (MQ=255)`  
                                                                                                                                                                                                                       `gtatcagagaaagttATGGACATTCCAGACGGTGCCAAGGATACTATGAAGTTGAAGTACCTTCAAGCGGACTCTAAGGACGGAGTATTCCGTACAGCCATCGGAACCATGGTCACTTACGCTGGTCGAGAGTGGTCTACCGCTGTGATTAACGGTAAGTTACCAGAACGAACCCCAGCTATGGATGCTCTGCGCAGAATCCGCAATGCTGACCCTCAGTTGATTGCTGCGCTATACCCAGACCAAGCTGAGCTATTCCTGACGATGGACATGATGGACAAGCAGGGTATTGCCCCTCAgg                                                                                      <  1:622993/287‑1 (MQ=255)`  
                                                                                                                                                                                                                        `cTGGGATTGACAGTATGGACATTCCAGACGGTGCCAAGGATGCTATGAAGTTGAAGTACCTTCAAGCGGACTCTAAGGGCGGAGCATTCCGTACAGCCATCGGAACCATGGTCACTGACGCTGGTCAAGAGTGGTCTGCCGCTGTGATTAACGGTAAGTTACCAGAACGAACCCCAGCTATGGATGCTCTGCGCAGAATCCGCAATGCTGACCCTCAGTTGATTGCTGCGCTATACCCAGACCAAGCTGAGCTATTCCTGACGATGGACATGATGGACAAGCAGGGTATTGACCCTCAGGt                                                                                     <  1:487027/301‑1 (MQ=255)`  
                                                                                                                                                                                                                        `cTGAGATTGACAGTATGGACATTCCAGACGGTGCCAAGGATGCTATGAAGTTGAAGTACCTTCACGCGGACTCTAAGGACGGAGTATTCCGTACAGCCATCGGAACCATGATCACTGACTCTGGTCAAGAGTGGTCTGCTGCTGTGATTAACGGTAAGTTACCAGAACGAACCCTAGCTATGGATGCTCTGCGCAGAATCCGCAATGCTGACCCTCCGTTGATTGCTGCGCTATACCCAGACTAAGCTGAGCTATTCCTCACGATGGACATGACGGTCAAGCAGGGTATTGACCCTCAGGt                                                                                     >  1:352274/1‑301 (MQ=255)`  
                                                                                                                                                                                                                          `gagaTTGACAGTATGGACATTCCAGACGGTGCCAAGGATGCTATGAAGTTGAAGTACCTTCAAGCGGACTCTAAGGACGGAGTATTCCGTACAGCCATCGGAACCATGGTCACTGACGCTGGTCAAGAGTGGTCTGCCGCTGTGATTAACGGTAAGTTACCAGAACGAACCCCAGCTATGGATGCTCTGCGCAGAATCCGCAATGCTGACCCTCAGTTGATTGCTGCGCTATACCCAGACCAAGCTGAGCTATTCCTGACGATGGACATGATGGACAAGCAGGGTATTGACCCTCAGGttt                                                                                   >  1:217683/1‑300 (MQ=255)`  
                                                                                                                                                                                                                             `aTTGACAGTATGGACATTCCAGACGGTGCCAAGGATGCTATGAAGTTGAAGTACCTTCAAGCGGACTCTAAGGGCGGAGCATTCCGTACAGCCATCGGAACCATGGTCACTGACGCTGGTCAAGAGTGGTCTGCAGCTGTGATTAACGGTAAGTTACCAGAACGAACCCCAGCTATGGATGCTCTGCGCAGAATCCGCAATGCTGACCCTCAGTTGATTGCTGCGCTATACCCAGACCAAGCTGAGCTATTCCTGACGATGGACATGATGGACAAGCAGGGTATTGACCCTCAGGTTATTc                                                                                <  1:162480/301‑1 (MQ=255)`  
                                                                                                                                                                                                                              `ttGACAGTATGGACATTCCAGACGGTGCCAAGGATGCTATGAAGTTGAAGTACCTTCAAGCGGACGCTAAGGACGGAGTATTCCGTACAGCCATCGGAACCATGGTCACTGACGCTGGTCAAGAGTGGTCTGCCGCTGTGATTAACGGTAAGTTACCAGAACGAACCCCAGCTATGGATGCTCTGCGCAGAATCCGCAGTGCTGACCCTCAGTTGATTGCTGCGCTATACCCAGACCAAGCTGAGCTATTCCTGACGATGGACATGATGGACAAGCAGGGTATTGACCCTCAGGTTATTCt                                                                               <  1:480766/301‑1 (MQ=255)`  
                                                                                                                                                                                                                               `tGACAGTATGGACATTCCAGACGGTGACAAGGATGCTATGAAGTTGAAGTACCTTCAAGCGGACTCTAAGGACGGAGCATTCCGTACAGCCATCGGAACCATGGTCACTGACGCTGGTCAAGAGTGGTCTGACGCTGTGATTAACGGTAAGTTACCAGAACGAACCCCAGCTATGGATGCTCTGCGCAGAATCCGCAATGCTGACCCTCAGTTGATTGCTGCGCTATACCCAGACCAAGCTGAGCTATTCCTGACGATGGACATGATGGACAAGCAGGGTATTGACCCTCAGGTTATTCtt                                                                              <  1:52938/301‑1 (MQ=255)`  
                                                                                                                                                                                                                                `gACAGTATGGACCTTCCAGACGGTTCCAAGGATGCTATGAAGTTGAAGTACCTTCAAGCGGACTCTAAGGACGGAGCATTCCGTACAGCCATCGGAACCATGGTCACTGACGCTGGTCAAGAGTGGTCTGCCGCTGTGATTAACTGTAAGTTACCAGAACGAACCCCAGCTATGGATGCTCTGCGCAGAAACCGCAATGCTGACCCTCAGTTGATTGCTGCGCTATACCCAGACCAAGCTGAGCTATTCCTGACGATGGACATGATGGACAAGCAGGGTATTGACCCTCAGGTTATTCTTg                                                                             <  1:222076/301‑1 (MQ=255)`  
                                                                                                                                                                                                                                    `gTATGGACATTCCAGACGGTGCCAAGGATGCTATGAAGTTGAAGTACCTTCAAGCGGACTCTAAGGACGGAGTATTCCGTACAGCCATCGGAACCATGGTCACTGACGCTGGTCAAGAGTGGTCTGCCGCTGTGATTAACGGTAAGTTACCAGAACGAACCCCAGCTATGGATGCTCTGCGCAGAATCCGCAATGCTGACCCTCAGTTGATTGCTGCGCTATACCCAGACCAAGCTGAGCTATTCCTGACGATGGACATGATGGACAAGCAGGGTATTGAcccctgtctcttatacccatt                                                                         >  1:263720/1‑283 (MQ=255)`  
                                                                                                                                                                                                                                    `cagtGGACATTCCAGACGGTGCCAAGGATGCTATGAAGTTGAAGTACCTTCAAGCGGACTCTAAGGACGGAGTATTCCGTACAGCCATCGGAACCATGGTCACTGACGCTGGTCAAGAGTGGTCTGCCGCTGTGATTAACGGTAAGTTACCAGAACGAACCCCAGCTATGGATGCTCTGCGCAGAATCCGCAATGCTGACCCTCAGTTGATTGCTGCGCTATACCCAGACCAAGCTGAGCTATTCCTGACGATGGACATGATGGACAAGCAGGGTATTGACCCTCAGGTTATTCTTGATGc                                                                         <  1:123880/298‑1 (MQ=255)`  
                                                                                                                                                                                                                                        `ggACATTCCAGACGGTGCCAAGGATGCTATGAAGTTGAAGTACCTTCAAGCGGACTCTAAGGACGGAGTATTCCGTACAGCCATCGGAACCATGGTCACTGACGCTGGTCAAGAGTGGTCTGCCGCTGTGATTAACGGTAAGTTACCAGAACGAACCCCAGCTATGGATGCTCTGCGCAGAATCCGCAATGCTGACCCTCAGTTGATTGCTGCGCTATACCCAGACCAAGCTGAGCTATTCCTGACGATGGACATGATGGACAAGCAGGGTATTGACCCTCAGGTTATTCTTGATGccgac                                                                     >  1:447876/1‑301 (MQ=255)`  
                                                                                                                                                                                                                                                      `gTGCCAAGGATGCTATGAAGTTGAAGTACCTTCAAGCGGACTCTAAGGACGGAGCATTCCGTACAGCCATCGGAACCATGGTCAATGACGCTGGTCAAGAGTGGTCTGCCGCTGTGATTAACGGTAAGTTACCAGAACGAACCCCAGCTATGGATGCTCTGCGCAGAATCCGCAATGCTGACCCTCAGTTGATTGCTGCGCTATACCCAGACCAAGCTGAGCTATTCCTGCCGATGGACATGATGGACAAGCAGGAGATTGACCCTACGGTTATTCTTGATGCCGACCGACGGACTGTTaa                                                       >  1:280745/1‑301 (MQ=255)`  
                                                                                                                                                                                                                                                       `ggCCAAGGATGCTATGAAGTTGAAGTACCTTCAAGCGGACTCTAAGGACGGGGTATTCCGTACAGCCATCGGAACCATGGTCACTGACGCTGGTCAAGAGTGGTCTGCCGCTGTGATTAACGGTAAGTTACCAGAACGAACCCCAGCTATGGATGCTCTGCGCAGAATCCGCAATGCTGACCCTCAGTTGATTGCTGCGCTATACCCAGACCAAGCTGAGATATTCCTGACGATGGACATGATGGACAAGCAGGGTATTGACCCTCAGGTTATTCTTGATGCCGACCGACTGACTATTAAg                                                      <  1:274281/300‑1 (MQ=255)`  
                                                                                                                                                                                                                                                                  `cTATGAAGTTGAAGTACCTTCAAGCGGACTCTAAGGACGGAGTATTCCGTACAGCCATCGGAACCATGGTCGCTGACGCTGTTCAAGAGTGGTCTGCCGCTGTGATTAACGGTAAGTTACCAGAACGAACCCCAGCTATGGATGCTCTGCGCAGAATCCGCAATGCTGACCCTCAGTTGATTGCTGCGCTATACCCAGACCAAGCTGAGCTATTCCTTACGATGGACAGGATGGACAAGCAGGGTATTGACCCTCAGGTTATTCTTGATGCCGACCGACTGACTGTTAAGCGGTCCAAaga                                           >  1:241642/1‑301 (MQ=255)`  
                                                                                                                                                                                                                                                                   `aaTGAAGTTGAAGTACCTTCAAGCGGACTCTAAGGACGGAGTATTCCGTACAGCAATCGGAACCATGGTCACTGACGCTGGTCAAAAGTGGTCTGCCGATGTGATTAACGGTAAGTTACCAGAACGAACCCCAGCTATGGATGCTCTGCGCAGAATCCGCAATGCTGACCCTCAGTTGATTGCTGCGCTATACCCAGACCAAGCTGAGCTATTCCTGACGATGGACATGATGGACAAGCAGGGTATTGACCCTCAGGTTATTCTTGATGCCGACCGACTGACTGTTAAGCGGTCCAAagag                                          <  1:46527/300‑1 (MQ=255)`  
                                                                                                                                                                                                                                                                    `aTGAAGTTGAAGTACCTTCAAGCGGACTCTAAGGACGGAGTATTCCGTACAGCCATCGGAACCATGGTCACTGACGCTGGTCAAGAGTGGTCTGCCGCTGTGATTAACGGTAAGTTACCAGAACGAACCCCAGCTATGGATGCTCTGCGCAGAATCCGCAATGCTGACCCTCAGTTGATTGCTGCGCTATACCCAGACCAAGCTGAGCTATTCCTGACGATGGACATGATGGACAAGCAGGGTATTGACCCTCAGGTTATTCTTGATGCCGACCGCCTGACTGTTAAGCGGTCCAAAGAGc                                         >  1:561598/1‑301 (MQ=255)`  
                                                                                                                                                                                                                                                                      `ataagagacagaCCTTCAAGCGGACTCTAAGGACGGAGTATTCCGTACAGCCATCGGAACCATGGTCACTGACGCTGGTCAAGAGTGGTCTGCCGCTGTGATTAACGGTAAGTTACCAGAACGAACCCCAGCTATGGATGCTCTGCGCAGAATCCGCAATGCTGACCCTCAGTTGATTGCTGCGCTATACCCAGACCAAGCTGAGCTATTCCTTACGATGGACATGATGGACAAGCAGGGTATTGACCCTCAGGTTATTCTTGATGCCGACCGACTGACTGTTAAGCGGTCCAAAGAGCaa                                       <  1:647084/290‑1 (MQ=255)`  
                                                                                                                                                                                                                                                                            `gaagtACCTTCAAGCGGACTCTAAGGACGGAGTATTCCGTACAGCCATCGGAACCATGGTCACTGACGCTGGTCAAGAGTGGTCTGCCGCTGTGATTAACGGTAAGTTACCAGAACGAACCCCAGCTATGGATGCTCTGCGCAGAATCCGCAATGCTGACCCTCAGTTGATTGCTGCGCTATACCCAGACCAAGCTGAGCTATTCCTGACGATGGACATGATGGACAAGCAGGGTATTGACCCTCAGGTTATTCTTGATGCCGACCGACTGACTGTTAAGCGGTCCAAAGAGCAAcctgtc                                 >  1:459215/1‑296 (MQ=255)`  
                                                                                                                                                                                                                                                                                 `aCCTTCAAGCGGACCCTAAGGGCGGAGCATTCCGTACAGCCATCGGAACCATGGTCACTGACGCTGGTCAAGAGTGGTCTGCCGCTGTGATTAACGGTAAGTTACCAGAACGAACCCCAGCTATGGATGCTCTGCGCAGAATCCGCAATGCTGACCCTCAGTTGATTGCTGCGCTATACCCAGACCAAGCTGAGCTATTCCTGACGATGGACATGATGGACAAGCAGGGTATTGACCCTCAGGTTATTCTTGATGCCGACCGACTGACTGTTAAGCGGTCCAAAGAGCAACGCTTTGAGga                            <  1:541480/301‑1 (MQ=255)`  
                                                                                                                                                                                                                                                                                       `aaGCGGACTCTAAGGACGGAGGATTCCGTACAGCAATCGGAACCATGGTCACTGACGCTGGTCAAGAGTGGTCTTCCGCTGTGATTAACGGTAAGTTACCAGAACGAACCCCAGCTATGGATGCTCTGCGCAGAATCCGCAATGCTGACCCTCAGTTGATTGCTGCGCTATTCCCAGACCAAGCTGAGCTATTCCTGACGATGGACATGATGGACAAGCAGGGTATTGACCCTCAGGTTATTCTTGATGCCGACCGACTGACTGTTAAGCGGTCCAAAGAGCAACGCTTTGAGGATGATaa                      <  1:157517/301‑1 (MQ=255)`  
                                                                                                                                                                                                                                                                                            `gACTCTAAGGGCGGAGAATTCCGTACAGACATCGGAACCATGGTCACTGACGCTGGTCAAGAGTGGTCTGCCGCTGTGATTAACGGTAAGTTACCAGAACGAACCCCAGCTATGGATGCTCTGCGCAGAATCCGCAATGCTGACCCTCAGTTGATTGCTGCGCTATACCCAGACCAAGCTGAGCTATTCCTGACGATGGACATGATGGACAAGCAGGGTATTGACCCTCAGGTTATTCTTGATGCCGACCGACTGACTGTTAAGCGGTCAAAAGAGCAACGCTTTGAGGAGGATAACGCAt                 >  1:616964/1‑301 (MQ=255)`  
                                                                                                                                                                                                                                                                                               `tctAAGGACGGAGTATTCCGTACAGCCATCGGAACCATGGTCACTGACGCTGGTCAAGAGTGGTCTGCCGCTGTGATTAACGGTAAGTTACCAGAACGAACCCCAGCTATGGATGCTCTGCGCAGAATCCGCAATGCTGACCCTCAGTTGATTGCTGCGCTATACCCAGACCAAGCTGAGCTATTCCTGACGATGGACATGATGGACAAGCAGGGTATTGACCCTCAGGTTATTCTTGATGCCGACCGACTGACTGTTAAGCGGTCCAAAGAGCAACGCTTTGAGgatgatctgtctctta              >  1:398459/1‑291 (MQ=255)`  
                                                                                                                                                                                                                                                                                                    `ggACGGAGCATTCCGTACAGCCATCGGAACCATGGTCACTGACGCTGGTCAAGAGTGGACTGCCGCTGTGATTAACGGTAAGTTACCAGAACGAACCCCAGCTATGGATGCTCTGCGCAGAATACGCAATGCTGACCATAAGTTGATTGCTGCGCTATACCCAGACCAAGCAGAGCTATTCCTGACGACGGACATGATGGACAAGCAGGGTATTGACCCTCAGGTTATTCTTGATGCCGACGGTCTGACGGTTAAGCGGTGCAAAGAGCAACGCTTTGAGGATGATTAAGGATTGGAGttg         >  1:363679/1‑299 (MQ=255)`  
                                                                                                                                                                                                                                                                                                        `ggAGTATTCCGTACAGCCATCGGAACCATGGTCACTGACGCTGGTCAAGAGTGGTCTGCCGCTGTGATTAACGGTAAGTTACCAGAACGAACCCCAGCTATGGATGCTCTGCGCAGAATCCGCAATGCTGACCCTCAGTTGATTGCTGCGCTATACCCAGACCAAGCTGAGCTATTCCTGACGATGGACATGATGGACAAGCAGGGTATTGACCCTCAGGTTATTCTTGATGCCGACCGACTGACTGTTAAGCGGTCCAAAGAGCAACGCTTTGAGGATGATAAAGCATTCGAGTCTGCAc     <  1:188859/301‑1 (MQ=255)`  

                                                                                                                                                                                                                                                                                                            `|`                                                                                                                                                                                                                                                                                                             
`AAGTTCAGAATCAGATGAACGCATGGACGAAAGCTCAGGCCAAGGCTCTGGACGATTCCATGAAGTCAATGAACAAACTTGACGTAATCGACAAGCAATTCCAGAAGCGAATCAACGGTGAGTGGGTCTCAACGGATTTTAAGGATATGCCAGTCAACGAGAACACTGGTGAGTTCAAGCATAGCGATATGGTTAACTACGCCAATAAGAAGCTCGCTGAGATTGACAGTATGGACATTCCAGACGGTGCCAAGGATGCTATGAAGTTGAAGTACCTTCAAGCGGACTCTAAGGACGGAGCATTCCGTACAGCCATCGGAACCATGGTCACTGACGCTGGTCAAGAGTGGTCTGCCGCTGTGATTAACGGTAAGTTACCAGAACGAACCCCAGCTATGGATGCTCTGCGCAGAATCCGCAATGCTGACCCTCAGTTGATTGCTGCGCTATACCCAGACCAAGCTGAGCTATTCCTGACGATGGACATGATGGACAAGCAGGGTATTGACCCTCAGGTTATTCTTGATGCCGACCGACTGACTGTTAAGCGGTCCAAAGAGCAACGCTTTGAGGATGATAAAGCATTCGAGTCTGCACTGA  >  NC_001604.1/29397‑29996`

  

Alignment Legend

Aligned base mismatch/match (shaded by quality score): `ATCG/ATCG` `< 3 ≤` `ATCG/ATCG` `< 10 ≤` `ATCG/ATCG` `< 22 ≤` `ATCG/ATCG` `< 34 ≤` `ATCG/ATCG` `< 38 ≤` `ATCG/ATCG`

Unaligned base: `atcg`    Masked matching base: `atcg`    Alignment gap: `‑`    Deleted base: `‑`
